# Frequency and clinical significance of Herpes simplex virus type 1/2 reactivation in adult patients with mild to moderately severe community-acquired pneumonia: a multicentre cohort study

**DOI:** 10.1007/s15010-024-02351-5

**Published:** 2024-07-20

**Authors:** Christina Bahrs, Christian Schönherr, Marcus Panning, Norman Rose, Theo Dähne, Stefan Hagel, Sebastian Weis, Jan Rupp, Gernot Rohde, Martin Witzenrath, Mathias W. Pletz, the CAPNETZ Study Group

**Affiliations:** 1 Institute for Infectious Diseases and Infection Control, Jena University Hospital– Friedrich Schiller University, Am Klinikum 1, 07747 Jena, Germany; 2 Department of Medicine I, Division of Infectious Diseases and Tropical Medicine, Medical University of Vienna, Vienna, Austria; 3 Department of Medicine V, Department of Pneumology, Jena University Hospital– Friedrich Schiller University, Jena, Germany; 4 Faculty of Medicine, Institute of Virology, Medical Center–University of Freiburg, Freiburg, Germany; 5 Leibniz Institute for Natural Product Research and Infection Biology, Hans Knöll Institute-HKI, Jena, Germany; 6 Department of Infectious Diseases and Microbiology, University Hospital Schleswig-Holstein, Lübeck, Germany; 7 German Center for Infection Research (DZIF), Partner Site Hamburg-Lübeck-Borstel-Riems, Lübeck, Germany; 8 CAPNETZ STIFTUNG, Hannover, Germany; 9 Department of Respiratory Medicine, Goethe University, Frankfurt, University Hospital, Medical Clinic I, Frankfurt/Main, Germany; 10 Biomedical Research in Endstage and Obstructive Lung Disease Hannover (BREATH), German Center for Lung Research (DZL), Hannover, Germany; 11 Department of Infectious Diseases, Respiratory Medicine and Critical Care, Charité-Universitätsmedizin Berlin, Berlin, Germany

**Keywords:** Herpes simplex virus, Community-acquired pneumonia, CRB-65 score, Oxygen therapy, Hospital recovery scale

## Abstract

**Purpose:**

This study assessed the frequency, clinical significance, and risk factors for Herpes simplex virus (HSV) reactivation in immunocompetent patients with community-acquired pneumonia (CAP).

**Methods:**

The study included adult CAP-patients who were enrolled in the CAPNETZ study between 2007 and 2017 and had a residual sputum sample available for analysis. In addition to routine diagnostics, sputum and blood samples were tested for HSV-1/2 using PCR. Demographics, comorbidities, and CRB-65 score were compared between HSV-positive and negative patients using Fisher exact or Mann Whitney test. Logistic regression analyses investigated the influence of HSV reactivation on a modified hospital recovery scale (HRS) until day 7, divided into 3 categories (no oxygen therapy, oxygen therapy, ICU admission or death).

**Results:**

Among 245 patients, HSV-1 and HSV-2 were detected in 30 patients (12.2%, 95%CI 8.7–16.9) and 0 patients, respectively. All HSV-positive patients were hospitalized, had a CRB-65 severity score of 0–2 and survived the first 28 day. In the HSV-positive group, patients had a non-significantly higher median age (70.5 versus 66 years) and a higher rate of oncological comorbidities (16.7% versus 8.8%) compared to the HSV-negative group. Distribution of co-pathogens and outcome parameters did not significantly differ between both groups. In a multivariate logistic regression model, age (AOR 1.029, *p* = 0.012) and CRB-65 score (AOR 1.709, *p* = 0.048), but not HSV-1 as single or co-pathogen were independently associated with higher HRS.

**Conclusion:**

Our study suggests that HSV-1 reactivation is common in CAP but might not be associated with specific risk factors or a complicated disease course.

## Introduction

Estimated 80% of the population is latently infected with Herpes simplex virus (HSV) type 1 (HSV-1) or type 2 (HSV-2) [1]. Recent studies have shown that HSV-1 reactivation in the respiratory tract is common among patients with ventilator-associated pneumonia (VAP) [2, 3] and is associated with higher mortality rates [3, 4]. A recent meta-analysis by Hagel et al., which included data from one RCT and eight cohort studies found that acyclovir treatment was associated with significantly improved survival in mechanically ventilated patients with HSV reactivation in the respiratory tract [5]. In contrast to VAP, little is known about HSV-1 reactivation in mild to moderately severe community-acquired pneumonia (CAP). Therefore, we assessed the frequency, clinical significance, and risk factors for HSV reactivation in this patient cohort.

## Methods

In our study, we included all patients who were enrolled into the CAPNETZ study between 2007 and 2017 by 13 different centres in Germany and had a residual sputum sample available for HSV-testing. The CAPNETZ study (German Clinical Trials Register: DRKS00005274) is a prospective multicentre study on adults with at least one clinical symptom and radiologically confirmed CAP. Exclusion criteria were a hospital stay within the last 28 days, active tuberculosis, and severe immunosuppression, such as corticosteroid therapy of at least 20 mg prednisolone-equivalent per day for more than 14 days, as described previously [6]. For the current analysis, in addition to routine diagnostics (respiratory and blood culture, pneumococcal and legionella urine antigen tests), bio-banked sputum samples and EDTA blood samples were sent to the National Reference Laboratory for HSV at the University Hospital of Freiburg, Germany to perform PCR for HSV-1/2 (using AltoStar® HSV PCR Kit 1.5, altona Diagnostics GmbH Germany), a multiplex-PCR for viral pathogens (PCR FTD Respiratory Pathogens 21 Assay, Siemens Healthineers, Eschborn, Germany) and in house-PCRs for *Bordetella pertussis, Legionella pneumophila* and *Mycoplasma pneumoniae*. The CAPNETZ case report form records more than 150 clinical parameters, such as demographics, comorbidities, CRB-65 score [7], clinical signs and symptoms, laboratory values, hospital admission, length of hospitalization, need for oxygen therapy, vasopressors, mechanical ventilation, and ICU admission, as well as 28-day and 180-day mortality rates. To evaluate early outcome, we also calculated the maximum hospital recovery scale (HRS) [8] until day 7. For statistical analysis, we estimated the incidence of HSV reactivation by a relative frequency of cases with the PCR-confirmed pathogen (in sputum or blood) among all cases. Characteristics of patients were described as median (interquartile range) or number (percentage). We report 95% Wilson-score confidence interval for binominal proportions and simultaneous 95% confidence intervals for multinominal proportions. To identify risk factors for HSV-1 reactivation, baseline parameters of HSV-1 positive and negative patients were compared with Fisher exact test for categorical and Mann Whitney test for continuous variables. Additionally, we investigated the influence of HSV-1 as single pathogen and as co-pathogen, age, BMI, malignant disease, kidney disease, respiratory comorbidity and CRB-65 score on a modified HRS, divided into three categories (category 1 = no oxygen therapy, category 2 = oxygen therapy, category 3 = ICU admission or death) using ordinal logistic regression analyses.

## Results

Sputum samples of 245 patients and blood samples of 80 patients were included in this study. HSV-1 was identified in 30 patients (12.2%, 95%CI 8.7–16.9). Of these cases, 29 were detected in sputum samples and 1 in blood samples. All samples were tested negative for HSV-2 (0%, 95%CI 0.0–1.5). Among the 30 patients with HSV-1 detection, HSV was the only pathogen in 11 patients (36.7%, 95%CI 21.9–54.5). The remaining 19 patients (63.3%, 95%CI 45.5–78.1) had additional viral pathogens (*n* = 10) and/or bacterial pathogens (*n* = 13), and/or infection with *Aspergillus fumigatus* (*n* = 1).

All HSV-positive patients were hospitalized and had CAP of mild to moderate severity based on a CRB-65 score of 0–2. In the HSV-group, patients had a higher median age of 70.5 years compared to 66 years in the HSV-negative group. Additionally, the median BMI was higher in the HSV-group, 27 kg/m^2^ compared to 24.7 kg/m^2^ in the HSV-negative group. Underlying malignant neoplastic or chronic kidney diseases were both more common among patients with HSV detection (> 13%) than patients without HSV detection (< 9%), but we were unable to identify any significant risk factors associated with HSV-1 reactivation (see Table 1). We did not detect significant differences in pathogen distribution between the two patient groups. Rhinovirus remained the most common viral pathogen, while *Streptococcus pneumoniae* was the most common bacterial pathogen in both groups. Notably, influenza virus and RSV were not found in the HSV-group.

**Table 1:** Baseline parameters (demography, severity of disease, clinical characteristics, comorbidities and detected pathogens) of patients with community-acquired pneumonia (CAP) stratified by HSV-1 detection

Variable	CAP-patients with HSV-detection *n* = 30	CAP-patients without HSV-detection *n* = 215	Univariate *p*-value ^a^
Male sex	18 (60.0%, 95%CI 42.3–75.4)	146 (67.9%, 95%CI 61.4–73.8)	*p* = 0.411
Age in years (Median, IQR)	70.5 (59.3–78.0)	66.0 (52.0–75.0)	*p* = 0.128
Body mass index (Median, IQR)	27.0 (23.3–30.0)	24.7 (21.3–28.9)	*p* = 0.219
Nursing home resident	0 (0%, 95%CI 0.0–11.4)	6 (2.8%, 95%CI 1.3–6.0)	*p* = 1.0
Chronically bedridden	0 (0%, 95%CI 0.0–11.4)	2 (0.9%, 95%CI 0.3–3.3)	*p* = 1.0
CRB-65 Score (Median, IQR)	1 (0–1)	1 (0–1)	*p* = 0.822
CRB-65 Score			
0 (Mild CAP)	9 (30.0%, 95%CI 16.7–47.5)	68 (32.5%, 95%CI 26.3–39.3)	
1–2 (Moderate CAP)	21 (70.0%, 95%CI 56.7–87.5)	136 (65.1%, 95%CI 58.9–71.9)	
3–4 (Severe CAP)	0 (0.0%, 95%CI 0.0–17.5)	5 (2.4%, 95%CI 0.0–9.2)	
Clinical symptoms/signs at baseline			
Cough	28 (93.3%, 95%CI 78.7–98.2)	201 (93.5%, 95%CI 89.4–96.1)	*p* = 1.0
Purulent sputum	19 (63.3%, 95%CI 45.5–78.1)	160 (74.4%, 95%CI 68.2–79.8)	*p* = 0.271
Pathologic lung auscultation	28 (93.3%, 95%CI 78.7–98.2)	180 (83.7%, 95%CI 78.2–88.1)	*p* = 0.274
Fever	20 (66.7%, 95%CI 48.8–80.8)	132 (61.4%, 95%CI 54.7–67.6)	*p* = 0.689
Dyspnoea	21 (70.0%, 95%CI 52.1–83.3)	162 (75.3%, 95%CI 69.2–80.6)	*p* = 0.509
Respiratory rate per minute (Median, IQR)	22 (19–24)	20 (18–24)	*p* = 0.743
Oxygen saturation in % at baseline (Median, IQR)	93 (89.3–96)	92 (89–95)	*p* = 0.626
Hospital admission	30 (100%, 95%CI 88.6–100.0)	196 (91.2%, 95%CI 86.6–94.3)	*p* = 0.139
Vaccination against influenza within the last 12 months	12 (40.0%, 95%CI 24.6–57.7)	84 (39.1%, 95%CI 32.8–45.7)	*p* = 1.000
Vaccination against pneumococci with the last 5 years	3 (10.0%, 95%CI 3.5–25.6)	21 (9.8%, 95%CI 6.5–14.5)	*p* = 1.000
Inhaled corticosteroids (previous therapy > 2 weeks) ^b^	4/19 (21.1%, 95%CI 8.5–43.3)	47/179 (26.3%, 95%CI 20.4–33.2)	*p* = 0.786
Oral continuous therapy with corticosteroid > 2 weeks ^b^	0/19 (0%, 95%CI 0.0–16.8)	12/179 (6.7%, 95%CI 3.9–11.4)	*p* = 0.610
Comorbidities			
Respiratory tract	12 (40.0%, 95%CI 24.6–57.7)	101 (47.0%, 95%CI 40.4–53.6)	*p* = 0.559
COPD	7 (23.3%, 95%CI 11.8–40.9)	70 (32.6%, 95%CI 26.6–39.1)	*p* = 0.402
Long term oxygen therapy	0 (0.0%, 95%CI 0.0–11.4)	25 (11.6%, 95%CI 8.0–16.6)	*p* = 0.052
Cardiac insufficiency	4 (13.3%, 95%CI 5.3–29.7)	31 (14.4%, 95%CI 10.3–19.7)	*p* = 1.000
Other cardiac disease	10 (33.3%, 95%CI 19.2–51.2)	75 (34.9%, 95%CI 28.8–41.5)	*p* = 1.000
Solid malignant disease	5 (16.7%, 95%CI 7.3–33.6)	19 (8.8%, 95%CI 5.7–13.4)	*p* = 0.189
Liver	0 (0.0%, 95%CI 0.0–11.4)	7 (3.3%, 95%CI 1.6–6.6)	*p* = 0.602
Kidney	4 (13.3%, 95%CI 5.3–29.7)	17 (7.9%, 95%CI 5.0–12.3)	*p* = 0.302
Cerebrovascular	0 (0.0%, 95%CI 0.0–11.4)	9 (4.2%, 95%CI 2.2–7.8)	*p* = 0.606
Neurological	1 (3.3%, 95%CI 0.6–16.7)	8 (3.7%, 95%CI 1.9–7.2)	*p* = 1.000
Detected pathogens other than HSV	19 (63.3%, 95%CI 45.5–78.1)	121 (56.3%, 95%CI 49.6–62.7)	*p* = 0.556
Viral pathogens	10 (33.3%, 95%CI 19.2–51.2)	77 (35.8%, 95%CI 29.7–42.4)	*p* = 0.842
Influenza virus A/B	0 (0.0%, 95%CI 0.0–11.4)	20 (9.3%, 95%CI 6.1–13.9)	*p* = 0.145
Parainfluenza virus	2 (6.7%, 95%CI 1.8–21.3)	7 (3.3%, 95%CI 1.6–6.6)	*p* = 0.304
RSV	0 (0.0%, 95%CI 0.0–11.4)	6 (2.8%, 95%CI 1.3–6.0)	*p* = 1.000
Rhinovirus	8 (26.7%, 95%CI 14.2–44.4)	29 (13.5%, 95%CI 9.6–18.7)	*p* = 0.097
Enterovirus	1 (3.3%, 95%CI 0.6–16.7)	1 (0.4%, 95%CI 0.1–2.6)	*p* = 0.230
Other viruses ^c^	0 (0.0%, 95%CI 0.0–11.4)	17 (7.9%, 95%CI 5.0–12.3)	*p* = 0.239
Bacterial pathogens	13 (43.3%, 95%CI 27.4–60.8)	68 (31.6%, 95%CI 25.8–38.1)	*p* = 0.218
* Streptococcus pneumoniae*	7 (23.3%, 95%CI 11.8–40.9)	23 (10.7%, 95%CI 7.2–15.5)	*p* = 0.069
* Staphylococcus aureus*	1 (3.3%, 95%CI 0.6–16.7)	5 (2.3%, 95%CI 1.0–5.3)	*p* = 0.547
* Haemophilus influenzae*	1 (3.3%, 95%CI 0.6–16.7)	9 (4.2%, 95%CI 2.2–7.8)	*p* = 1.000
* Mycoplasma pneumoniae*	1 (3.3%, 95%CI 0.6–16.7)	18 (8.4%, 95%CI 5.4–12.8)	*p* = 0.483
* Legionella pneumophila*	0 (0.0%, 95%CI 0.0–11.4)	6 (2.8%, 95%CI 1.3–6.0)	*p* = 1.000
Enterobacterales	2 (6.7%, 95%CI 1.8–21.3)	7 (3.3%, 95%CI 1.6–6.6)	*p* = 0.304
Other bacteria ^d^	1 (3.3%, 95%CI 0.6–16.7)	4 (1.9%, 95%CI 0.7–4.7)	*p* = 0.483
Fungal pathogens	1 (3.3%, 95%CI 0.6–16.7)	2 (0.9%, 95%CI 0.3–3.3)	*p* = 0.325
* Aspergillus fumigatus*	1 (3.3%, 95%CI 0.6–16.7)	2 (0.9%, 95%CI 0.3–3.3)	p*p* = 0.325

Data are presented as a number (%, 95%CI) or the median together with the interquartile ranges (Q1–Q3)

^a^Patients with HSV detection were compared to patients without HSV detection using the Fisher exact test for categorical variables and the Mann–Whitney test for continuous variables. The significance level was set at a two-sided p-value ≤ 0.05

^b^Data collection on inhaled or oral corticosteroid therapy began on August 1, 2010

^c^Other viruses included adenovirus, bocavirus, coronavirus 229 E/NL 46/NL 63/OC43, human metapneumovirus, parechovirus

^d^Other bacteria included *Mycobacterium avium* in HSV-positive patients and *Pseudomonas aeruginosa, Stenotrophomonas maltophilia* in HSV-negative patients

All HSV-positive patients received antibiotic treatment, and only one patient with a co-infection with parainfluenza virus received acyclovir. For outcome comparison, we excluded 11 patients from the non-HSV group with missing data for the CRB-65 score (*n* = 6) or a score > 2 (*n* = 5). Within the first seven days, there was no significant difference in the maximum HRS between HSV-positive and HSV-negative patients (*p* = 0.398). The median HRS was 3 (range 2–4) in the HSV-group, compared to a median of 3 (range 1–6) in the non-HSV group. As shown in Table 2, the univariate analysis revealed no significant association (*p* ≥ 0.05) between a modified HRS (as mentioned above) and the presence of HSV-1 as single pathogen (*p* = 0.483) or HSV-1 as co-pathogen (*p* = 0.774). However, we found an association between a higher modified HRS and older age (*p* < 0.001), higher BMI (*p* = 0.044), higher CRB-65 score on admission (*p* < 0.001), and a respiratory comorbidity (*p* = 0.044). In the multivariate ordinal logistic regression analysis, only age (AOR 1.029, *p* = 0.012) and CRB-65 score (AOR 1.709, *p* = 0.048) remained independent predictors of the modified HRS. The median length of hospital stay was 9 days for both groups (*p* = 0.984). After 15 days, the clinical status was improved in 93.1% of the HSV-group compared to 80.7% in the non-HSV group (*p* = 0.123). Among the 30 HSV-positive patients, only one (3.3%) was admitted to ICU, but without the need of mechanical ventilation or vasopressors. In contrast, 14 patients (6.9%, *p* = 0.486) of the 204 HSV-negative patients with a CRB-65 score ≤ 2 at baseline were admitted to ICU, with 3 patients receiving vasopressors and 4 patients requiring mechanical ventilation. All HSV-positive patients survived the first 28 days, while 2 HSV-negative patients (1%) died within the first 7 days. The 180-day mortality rate in our study was low, with 3.3% in the HSV-positive and 3.4% in the HSV-negative group (*p* = 1.0).

**Table 2:** Univariate and multivariate analyses of the influence of HSV-1 as single pathogen, HSV-1 as co-pathogen, age, BMI, malignant comorbidity, renal comorbidity, respiratory comorbidity and CRB-65 score in patients with mild to moderate community-acquired pneumonia on the outcome modified Hospital Recovery Scale (HRS) within the first 7 days

Variable			OR (95%CI)	AOR (95%CI)
HSV-1 as single pathogen	YES (*N* = 11)	No detection of HSV (*N* = 204)		
Category 1:	1 (9.1%, 95%CI 0.0–24.2)	55 (27.0%, 95%CI 20.6–33.6)	1.572 (0.462–5.696)	1.330 (0.360–5.162)
Category 2:	10 (90.9%, 95%CI 81.8–100.0)	134 (65.7%, 95%CI 59.3–72.3)		
Category 3:	0 (0.0%, 95% CI 0.0–15.1)	15 (7.4%, 95%CI 1.0–14.0)		
HSV-1 as co-pathogen	YES (*N* = 19)	No detection of HSV (*N* = 204)		
Category 1:	4 (21.1%, 95%CI 5.3–40.4)	55 (27.0%, 95%CI 20.6–33.6)	1.156 (0.440–3.200)	1.034 (0.361–3.025)
Category 2:	14 (73.7%, 95%CI 57.9–93.1)	134 (65.7%, 95%CI 59.3–72.3)		
Category 3:	1 (5.3%, 95%CI 0.0–24.6)	15 (7.4%, 95%CI 1.0–14.0)		
Age in years	Median	IQR	1.047 (1.030–1.066)	1.029 (1.007–1.053)
Category 1: *N* = 60	53.5	39–67.5		
Category 2: *N* = 159	69.0	57–77		
Category 3: *N* = 16	70.5	63.5–75.8		
BMI	Median	IQR		
Category 1: *N* = 60	22.4	20.3–27	1.049 (1.002–1.099)	1.045 (0.996–1.098)
Category 2: *N* = 156	25.8	21.7–29.3		
Category 3: *N* = 16	25.8	24.3–34.5		
Malignant comorbidity	YES (*N* = 23)	NO (*N* = 212)		
Category 1:	3 (13.0%, 95%CI 4.3–30.1)	57 (26.9%, 95%CI 20.8–33.5)	1.614 (0.657–4.131)	0.975 (0.345–2.811)
Category 2:	19 (82.6%, 95%CI 73.9–99.7)	140 (66.0%, 95%CI 59.9–72.6)		
Category 3:	1 (4.3%, 95%CI 0.0–21.4)	15 (7.1%, 95%CI 0.9–13.7)		
Renal Comorbidity	YES (*N* = 21)	NO (*N* = 214)		
Category 1:	3 (14.3%, 95%CI 0.0–31.9)	57 (26.6%, 95%CI 20.6–33.1)	1.889 (0.720–5.160)	1.302 (0.457–3.800)
Category 2:	16 (76.2%, 95%CI 61.9–93.8)	143 (66.8%, 95%CI 60.7–73.3)		
Category 3:	2 (9.5%, 95% CI 0.0–27.2)	14 (6.5%, 95%CI 0.5–13.1)		
Respiratory comorbidity	YES (*N* = 111)	NO (*N* = 124)		
Category 1:	20 (18.0%, 95%CI 10.8–26.0)	40 (32.3%, 95%CI 24.2–41.5)	1.763 (1.021–3.082)	1.370 (0.763–2.478)
Category 2:	84 (75.7%, 95%CI 68.5–83.6)	75 (60.5%, 95%CI 52.4–69.7)		
Category 3:	7 (6.3%, 95%CI 0.0–14.3)	9 (7.3%, 95%CI 0.0–16.5)		
CRB-65 score	Median	IQR		
Category 1: *N* = 60	0	0–1	2.698 (1.782–4.157)	1.709 (1.009–2.925)
Category 2: *N* = 159	1	0–1		
Category 3: *N* = 16	2	1–2		

Category 1 (HRS ranging from 1 to 2) = no oxygen therapy, Category 2 (HRS of 3) = non-ICU but oxygen therapy, Category 3 (HRS ranging from 4 to 6) = ICU admission ± mechanical ventilation or death

## Discussion

The findings of this study indicate that HSV-1 reactivation is prevalent not only in invasively ventilated COVID-19 patients [4, 9], but also in patients with mild to moderately severe CAP, although to a lesser degree. In the present study, HSV-1, not HSV-2, was detected in sputum of more than 10% of immunocompetent patients with non-severe CAP. Similarly, a prospective investigation conducted over a decade ago in Turin, Italy, involving 237 hospitalized adult patients with respiratory symptoms, new pulmonary infiltrates, or routine follow-up in lung transplant recipients, revealed a high rate of HSV-1 positive BAL specimens (32.1%, 95%CI 26.5–38.3), in contrast to a low rate of HSV-2 positive samples (0.9%, 95%CI 0.3–3.1) [10]. Interestingly, this study did not find any association between immune (transplant) status, comorbidities or age and HSV-1 positivity. These results are consistent with our own research findings, as we were unable to identify specific risk factors for HSV-1 reactivation in CAP. However, in the study by Costa et al., a high viral load ≥ 10^5^ copies/ml was significantly associated with a complicated disease course, including admission to ICU, mechanical ventilation, and 28-day mortality [10]. In our study, an increasing HRS within the first seven days, with includes factors such as hospitalization, oxygen therapy, admission to ICU, mechanically ventilation, and mortality was associated with older age, higher BMI, respiratory comorbidities, and a higher CRB-65 score. But we did not find an association between worse outcome and HSV-1 as single pathogen or co-pathogen. Another recent study from Italy, focusing on 120 critically ill patients with severe COVID-19 pneumonia, reported even higher incidences of HSV reactivation (48% for HSV-1, 2.5% for HSV-2) [9]. In that study, all COVID-19 critically ill patients received systemic corticosteroid therapy following WHO guidelines. In contrast, our study involved less severely ill patients without SARS CoV-2 infection, with a low rate of prior oral glucocorticoid therapy of 6.1%.

It is important to note that only one case of HSV-positive patient received acyclovir treatment in our study. But it is crucial to emphasize that there are no recommendations for acyclovir therapy in immunocompetent patients with pneumonia, except for long-term mechanically ventilated patients with high HSV-1 viral loads in BAL samples [5].

The present study has a relevant number of limitations including the limited sample size, the use of sputum instead of tracheal aspirate or BAL, and the testing of only one sputum sample per patient without quantifying the viral load. Furthermore, we have not documented the presence of labial herpes, raising the possibility that the detection of HSV in some samples may have originated from facial sources rather than the respiratory tract. Despite these limitations, sputum remains a practical and non-invasive microbiological sample for use in non-critically ill patients, making it a common choice in the clinical settings. Our study aimed to provide a preliminary exploration into the incidence and clinical significance of HSV-1 in CAP. Larger prospective studies utilizing more invasive sampling methods are necessary to validate our findings and further investigate HSV reactivation in this context.

## Data Availability

The data sets used and/or analysed during the current study are available upon reasonable request from the corresponding author.
